# Clinical Application of Standardized Cognitive Assessment Using fMRI. II. Verbal Fluency

**DOI:** 10.3233/BEN-2008-0224

**Published:** 2009-07-29

**Authors:** Mark D. Allen, Alina K. Fong

**Affiliations:** ^1^Psychology DepartmentBrigham Young UniversityProvoUTUSA; ^2^Neuroscience CenterBrigham Young UniversityProvoUTUSA

**Keywords:** fMRI, verbal fluency, cognitive assessment, normative data

## Abstract

In this study, we describe an fMRI version of the verbal fluency test. This is the second in a series of fMRI adaptations of classical neuropsychological tests, for which normative samples of functional activation have been collected from unimpaired control subjects and structured in a manner that makes individual patient evaluation possible in terms of familiar z-score distributions. This fMRI protocol is shown to have strong convergent validity with the FAS phonemic fluency test and to elicit activation patterns highly consistent with a large body of previous neuroimaging studies of verbal fluency. We also present a case study, in which we report concurrent data from a patient with selective deficits in verbal processing, using both conventional neuropsychological and fMRI approaches. These analyses reveal striking correspondences between the deficits present in this patient on cognitive performance tests and the equally selective patterns of deviation present in his fMRI data.

## 1. Introduction

Functional magnetic resonance imaging (fMRI) is becoming increasingly recognized for its potential in clinical applications [7,10,16]. One area of recent interest concerns the development of fMRI as a practical tool for the diagnostic and assessment of cognitive functioning – that is, that fMRI tests might be relied upon in much the same manner as conventional "paper-pencil" tests are relied upon as a routine cognitive assessment method [26,27,33,37,42].

In order to bring fMRI technology into the clinical realm, however, there are at least two major issues that must be addressed. The first issue relates to limitations in the way cognitive tasks can be performed in the MRI scanning environment. Although, with some degree of innovation, it is possible to design fMRI tests that engage practically any cognitive function one might imagine, there remain substantial physical limitations which prohibit exact replications of activities performed on many conventional neuropsychological assessments. In fact, the very activity perhaps most common to all conventional exams – using a pencil and paper – is not possible in an MRI scanner. In spite of this, we suggest that satisfactory solutions are possible for a wide range of assessment types. We cite, for example, an ingenious solution to the above paper-pencil limitation reported by Zakzanis et al. [42] for their adaptation of the Trail Making Test, in which they employ an MRI-compatible virtual stylus. Regardless of the specific approach one might apply to the problem of fMRI adaptation, though, we contend that exact replications need not be achieved in most cases. Rather, a good approximation using similar stimuli and response methods might well suffice, so long as we can be confident that the fMRI adaptation measures the same cognitive construct that the conventional test is purported to measure. Thus, we suggest that concurrent validity testing might be applied in order to compare a given fMRI adaptation to the particular conventional test or tests it aims to model, in much the same way that conventional tests are commonly evaluated with respect to one another.

ISSN 0953-4180/08/$17.00 © 2008 – IOS Press and the authors. All rights reserved

A second, and perhaps more important issue, however, is that there are currently no widely available normative data sets for fMRI-based cognitive testing protocols (see [10] for recent discussion). The lack of such norms poses an obvious problem for the interpretation of outcomes from any fMRI protocol, regardless of how well that protocol might be designed. As is true for any assessment approach, reliable information about expected outcomes is essential to the process of evaluating degree of normalcy in individual patients. In the case of fMRI, information about normal brain activation patterns associated with a given cognitive task is needed in order to determine normalcy of activation patterns observed in individual patients who are scanned while performing that task.

In a companion paper [43] we describe an fMRI adaptation of the Matrix Reasoning Test (the f-MRT), in which we address both of the critical issues described above, by applying convergent validity testing and extensive normative data analysis. Additionally, in that paper we describe an application of this protocol in the assessment of a sample patient suffering cognitive impairments following traumatic brain injury, where we highlight unique contributions that fMRI scanning offers as a supplement to conventional testing. In particular, we demonstrate that the f-MRT assessment provides rich information about the unique nature of a patient's deficit at the cortical level, which allows us to distinguish subtypes of functional brain impairments among individuals who otherwise perform with the same degree of accuracy on conventional versions of the MRT.

In this paper we describe the second of a series of six fMRI protocols developed in our laboratory with the potential for clinical application. The protocol described in this study, which we refer to as the *f-VFT*, is an fMRI adaptation of classic phonemic verbal fluency tests. Following the format of the f-MRT report in Allen and Fong [43], we will first describe the method for creating the f-VFT, including an evaluation of convergent validity with the conventional test it aims to model (i.e., the FAS paradigm [5,8,20,25,39,41]). Next we present functional activation data from 32 control subjects without brain impairment who were scanned using the f-VFT protocol. Finally, we apply the same method for assessing the reliability of activation patterns across subjects introduced in Allen and Fong [43] to the f-VFT, which provides the basis for a map of normative activation patterns for this exam. The normative brain map represents a distribution of activation values within functionally-defined brain regions of interest (ROI), which allows one to evaluate individual patient result profiles expressed in terms of a pattern of $z$-scores across regions.

Following the experimental portion of this study, we will present a practical application of the f-VFT. In that section, we describe conventional and fMRI testing outcomes of a single patient suffering traumatic brain injury. The primary objective of that section will be to demonstrate the discriminative power of the f-MRT and f-VFT protocols within a single patient, in a manner that parallels the conventional tests they model. The patient we describe provides a particularly good case-study for this objective, given that his cognitive deficits show a marked selectivity, with impairments limited almost exclusively to verbal processing skills. Thus, to the extent that this patient shows selective impairments on traditional methods of assessment, we expect the same degree of selectivity in deviations from normal patterns in his fMRI data.

## 2. fMRI study of the verbal fluency test

Verbal fluency is a classic neuropsychological measure of executive functioning within the domain language production in which subjects are instructed to generate as many words as possible in a limited amount of time while adhering to specific word restriction rules [20]. Phonemic, or letter, fluency is a popular variant of this paradigm in which subjects are asked to generate words beginning with a specified letter while avoiding repetitions, multiple variants of a single word, and proper nouns [6]. The letters F, A, and S have been associated with this task since its inception by Benton [5], on the grounds that these letters have a very high word-initial frequency [8]. As such, the FAS letter series, along with a standard 60-second time limit for each letter, is included in more assessment batteries and normative data studies than any other letters [20, 25,39,41].

Although the FAS paradigm is relatively simple, there are some challenges in adapting it for fMRI use. First, standard application of this test requires overt vocal responses. This creates head motion which usually far exceeds acceptable limits for accurate functional image acquisition and analysis. Although there have been novel attempts to circumvent this problem, using overt responses with "paced" paradigms [1,3,4,13,14], we find these solutions to be inadequate for our purposes. There are two main reasons for this. In the paced paradigm, the subject is not allowed to freely generate words at any time, but must instead generate a single word response only at designated response opportunities, typically every 4 to 6 seconds. Second, the total number of words a subject may generate is limited by a pre-designated number of response opportunities. Although activation patterns observed using overt-response paradigms usually resemble those from covert-response designs for some key brain areas [11], review studies indicate significant sources of additional activation for overt designs, where response pacing, in particular, elicits activity related to non-linguistic processes that go beyond the essential task, such as increased response inhibition, motor preparation, and sustained attention [4]. Following the majority of previous fMRI studies of verbal fluency, our design uses covert responses. An obvious limitation of the covert paradigm is the inability to monitor subject performance. We suggest this problem might be overcome, to some degree, by verifying performance on a parallel overt-response task administered outside of the scanner.

There are two further factors that limit straightforward adaptation of the FAS test for fMRI environments. Namely, it includes only three letters and subjects are given 60 seconds to respond to each letter. In terms of fMRI design, an experiment with three trials at a duration of 60 seconds each is far from optimal for accurate statistical analysis. A design with more trials (e.g., 8) and shorter trial durations (e.g., 10–20 seconds) would greatly increase the paradigm's power, efficiency, and signal to noise resolution [17,21,22,24]. Accordingly, we made two modifications for the f-VFT. First, we increased the total number of target letters to 8, adding B, M, H, G and L. These letters were chosen because they retain the same proportion of associative frequency counts as F, A, and S, as determined by Borkowski et al. [8]. Second, we reduced durations of the f-VFT to 18 seconds. In our opinion, these modifications do not alter the most fundamental properties of the FAS. In order to verify this assumption, we performed convergent validity tests (reported below) in which independent samples of neurologically impaired and unimpaired subjects were administered both the standard FAS test and the f-VFT, with both tests administered outside of the MRI scanner.

### 2.1. Previous fMRI studies of verbal fluency: Expected regions of activation for the f-VFT

It is reasonable to expect that activation data from the f-VFT for normal subjects replicate what has been found in previous fMRI studies of verbal fluency. In fact, verbal fluency has been among the most frequent cognitive processes to be examined by fMRI. A recent review and meta-analysis by Costafreda et al. [11] identified 22 fMRI experiments on verbal fluency which met the criteria for the specific purpose of their review alone. Of these, all but three used covert response paradigms. This and other review studies implicate the left inferior frontal gyrus (LIFG) as the most prominent and reliable neural region involved in verbal fluency, where *pars opercularis* is most likely critical for the phonological processing demands of this task and *pars triangularis* is most likely implicated in semantic retrieval and selection demands [2,11,15,37].

These, along with other prominent neuroimaging studies of phonemic fluency [9,12,18,19,29,23,31,32] report activation peaks that are consistent with a general neural model of lexical retrieval and selection defended by Thompson-Schill and colleagues [40]. This model specifies lexical retrieval mechanisms in the temporal lobe, particularly in the posterior regions, along with mechanisms of lexical selection that include frontal structures, such as the anterior cingulate and left inferior frontal gyrus. The above studies also support the role of the medial SMA and dorsal anterior cingulate as mechanisms for response sequence planning and response inhibition during verbal fluency performance, as well as portions of dorsolateral prefrontal cortex and frontal operculum, which are associated with executive functioning and attentional demands. Furthermore, activation is consistently found in left hemisphere speech production/articulation areas (inferior precentral gyrus and adjacent premotor cortex), even when covert responding is used.

### 2.2. Methods

#### 2.2.1. Participants

Thirty-two participants (16 male, 16 female) between 20 and 39 years old (Mean = 25.04; S.D = 4.23) volunteered to serve as control subjects for this study. Participants received no compensation, but were told that they would have the opportunity to see structural and functional images of their brains after the study. Hand dominance was assessed using the Edinburgh Handedness Inventory [28]. All but two subjects (one male, one female) were determined to be dominantly right-handed. Mean L.Q. scores on the Edinburgh handedness scale – where scores above +48 suggest strong right handedness – were +71.8 (Decile R.3), SD = 35.0 for females; and +69.1 (Decile R.3), SD = 31.5 for males; with no significant difference between sexes ($t = 1.36, p > 0.1$). All subjects were Caucasian, except for one Hispanic woman and one Asian/Pacific Islander male, and spoke English as their first language.

All participants were determined to have no history of neurological impairments (assessed by a screening questionnaire), nor history of significant psychological pathology, and reported no use of psychotropic medications. High resolution 3D SPGR and $T_2$ axial FLAIR MRI scans revealed no detectible brain abnormalities in any control subjects, as determined by a qualified neuroradiologist. In addition to the overall good neuropsychological health of our control participants, they were also determined to be high functioning in cognitive ability. All subjects had completed at least one year of college education and were in good academic standing at a university with high admission/continuance standards. All participants consented to release pre-admission records of ACT (or SAT) scores. Analysis of mean scores (with SAT converted to ACT equivalents) revealed overall high performance, with a mean of 30 (SD = 4.30) for females, and 29 (SD = 2.16) for males, with no significant difference between sexes ($t = 1.38$, $p > 0.1$).

*2.2.2. Materials*

The f-VFT includes a total of 8 target letter cues: F, A, S, B, M, H, G and L.

*2.2.3. Concurrent validity with the standard FAS test*

In order to assess correlations in performance outcomes between the f-VFT and the conventional FAS test applications, we collected additional data from a sample of 38 individuals without neurological impairment, as well as 17 individuals diagnosed with neurological/cognitive impairments. The 38 subjects without impairment were matched demographically to the participants in the fMRI study, in terms of age, sex, and education level. The 17 patients with neurological impairments included 6 women and 11 men with an age range of 22–85 years, who were referred for clinical fMRI scans. This sample included 1 individual diagnosed with vascular dementia, 4 with probable Alzheimer's Disease, and 12 with cognitive impairments following traumatic brain injury. All participants were administered both the FAS test and a non-scanner version of the f-VFT, with test order counterbalanced across participants. Instructions for both tests included the exclusion rules for standard administration of the FAS. In order to avoid practice/interference effects from repeating letter cues in both tests, only the 6 added letters B, M, H, G and L were included in this version of the f-VFT. This also limits potential artificial inflation of convergent validity due to partial stimulus overlap. Time limits for the FAS and f-VFT tests were 60 and 18 seconds, respectively.

*2.2.4. Unimpaired subjects*

For this sample, the mean error-corrected score on the standard FAS test was 47.1 (SD = 10.83) words. The mean error-corrected score for the f-VFT was 12.26 (SD = 2.98) words. Correlation analysis revealed a coefficient of +0.85, such that the FAS and f-VFT show strong concurrent/convergent validity in a sample of individuals without brain impairment.

*2.2.5. Neurologically impaired subjects*

Performance scores for this group of 17 subjects showed slightly more variability. The mean error-corrected score for the standard FAS test was 35.16 (SD = 13.10) words. The mean error-corrected score for the f-VFT was 11.29 (SD = 2.99) words. Correlation analysis revealed a coefficient of +0.89, suggesting that the f-MRT and CPM show strong concurrent/convergent validity in a sample of patients with a variety of neurological/cognitive impairments.

*2.2.6. fMRI testing procedures*

Prior to functional sessions, participants were given instructions about the task, including exclusion criteria, and given a practice trial with feedback using the letter T. All test stimuli were presented via MRI-compatible LCD goggles. At the beginning of each session, a "please wait prompt" appeared for 8 seconds to allow for T1 relaxation effects. This was followed by the prompt "Think of words that begin with the letter . . . " presented for 2 seconds. Immediately following the word generation prompt, a target letter cue was presented for 18 seconds. Subjects were instructed to covertly generate as many words as possible, according to exclusion rules, until the cue disappeared. Each 20 second test block, consisting of a word generation prompt followed by a letter cue, alternated with an 11-second "rest" block, in which subjects were instructed to count covertly from 1 to 10. This simple counting task is recommended as an optimal minimal-demand cognitive activity for "rest" epochs in fMRI experiments [38].

## 2.3. Data analysis

### 2.3.1. Image acquisition

Functional images were acquired at 23 contiguous axial locations with a slice thickness of 5 mm, using an EPIBOLD sequence with the critical parameters TR = 2000; TE = 40 ms. Conventional pre-processing and statistical analyses were performed using MRI-cro and SPM2 (http://www.fil.ion.ucl.ac.uk) software packages, respectively. Preprocessing procedures included acquisition time realignment, using sinc interpolation, followed by motion correction with EPI distortion unwarping. No head movement exceeded 1 mm translation or $1^\circ$ rotation displacement. After motion/distortion correction, all functional volumes were spatially normalized and resampled using the Montreal Neurological Institute (MNI) templates implemented in SPM2, and spatially smoothed with an 8mm FWHM Gaussian kernel, in order to increase signal-to-noise ratio and to reduce the effects of moderate intersubject variability in brain anatomy. A high-resolution 3D SPGR whole-head volume was also collected from each subject and examined by a neuroradiologist for any structural anomalies that might disqualify the participant as a "normal" control subject. Each subject's SPGR image was then coregistered and normalized to their mean functional image in order to perform subject-specific ROI analyses that take into account individual variability in cortical landmark organization.

### 2.3.2. Conventional fMRI analyses

#### 2.3.2.1. Subject-level analysis

A time-series ANCOVA implemented in SPM2 was used to test each voxel, for each subject, against the null-hypothesis that changes in BOLD signal in that voxel, over the duration of the experiment, did not significantly correlate with the temporal sequencing of the cognitive task of interest. A boxcar waveform convolved with a synthetic hemodynamic response function (HRF) with a 4 sec lag-to-peak was used to model task-related activation. The data were high-passed-filtered in time, using a set of discrete cosine basis functions with a cut-off period of 128 seconds, and conditioned for temporal autocorrelations using AR1 correction. For each participant, $t$-values for the contrast test condition versus control condition, as well as the simple contrast test condition (against an implicit baseline) were computed for each voxel, using the parameter estimates of the ANCOVA. The resulting 3-dimensional contrast map from each subject was saved for further subject-level ROI analysis as well as for random effects (RFX) group-level analysis.

#### 2.3.2.2. Group-level analysis

Activation at the group level was analyzed using the RFX approach recommended by Penny et al. [30], in which the value of the sum of the contrast weights for each voxel from each subject's ANCOVA was entered as a single data point in a *second-level* $t$-statistic computation, with the mean value for each voxel across subjects modeled as the effect term and the variance between subjects modeled as the error term. Significant activation peaks at the group-level are reported with a critical FWE corrected $p$-value of $< 0.001$, and a voxel cluster extent threshold of 8.

### 2.3.3. ROI analysis

In addition to the RFX group-level analysis, we performed ROI-based analyses for each control participant. There were two reasons for this: First, as stated above, a primary objective of this study is to develop a method for assessing the reliability of activation patterns across subjects, in order to provide quantitative estimates of deviation for any individual patient against a normative sample. For our approach, these analyses were carried out in terms of functionally motivated ROIs. Second, the RFX model is sensitive only to activation that reliably occurs across subjects within in a relatively tight spatial proximity. Therefore, for some brain regions, such as dorsolateral prefrontal cortex, the RFX model might not be sensitive to some locally idiosyncratic yet globally systematic patterns of activation.

Our procedure for establishing functional ROI designations and extracting activation values from individual subjects is described in detail in Allen and Fong [43]. Following the procedures described there, means and standard deviations of extracted scores were computed across subjects and used to derive a normalized distribution of $z$-scores for each ROI.

## 2.4. Results

### 2.4.1. Task performance on the f-VFT

Because responses were covert, it was not possible to record the number of words generated during on-line trials. The convergent validity study described above estimated that subjects produced an average of 12.26 words (SD = 2.98) per trial. It is important to note that during structured debriefing sessions all participants reported that they had been entirely compliant and had put forth genuine effort. We are fairly confident in these statements, as all control subjects displayed positive motivation by volunteering for our study and were interested in seeing their own activation results at the end of the study.

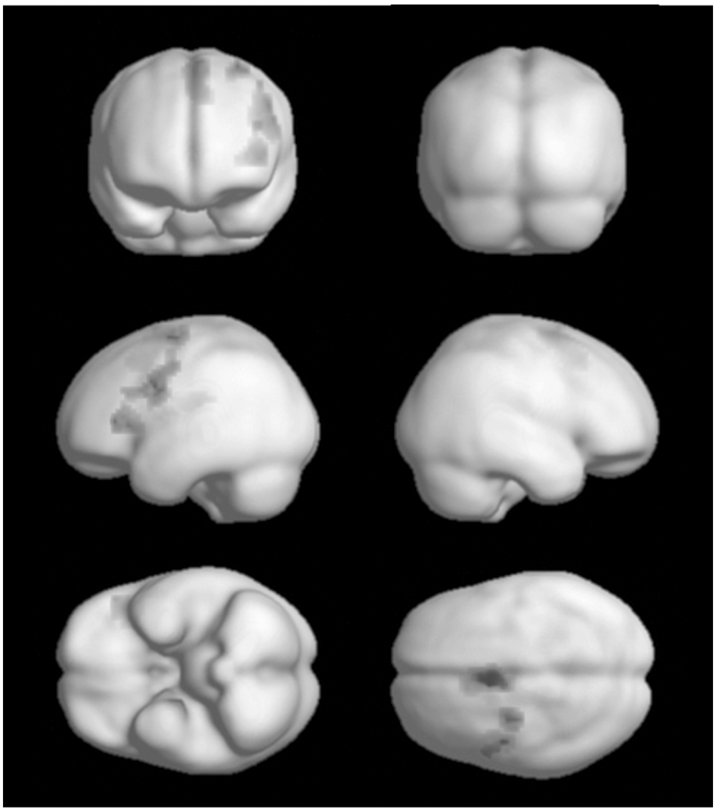

Fig. 1. Distribution of significant group-level activation for the f-VFT surface-projection rendered on smoothed MNI brain template.

*2.4.2. Group-level BOLD activation: RFX model*

Mean activation at the group level was highly consistent with patterns found in similar studies (Fig. 1). Results based on the contrast test-control differed only slightly from the simple contrast test (versus implicit baseline). Given our interest in whole brain activation associated with all perceptual and cognitive components of the VFT, we report the slightly more comprehensive pattern found for the simple contrast test. See Table 1a for a complete summary. As expected, activation was found almost exclusively in left hemisphere language areas associated with lexical form retrieval/selection, and covert articulation, including precentral/premotor cortex, inferior frontal gyrus/frontal operculum, basal ganglia, and thalamus. Consistent with previous studies, activation of medial SMA was also present. However, group-level activation was not found in two expected regions: dorsal anterior cingulate gyrus, which is associated with the selection and inappropriate response inhibition components of the task, and the posterior temporal lobe, which is associated with semantic retrieval components of this task. However, the ROI analyses reported below revealed reliable activation in these areas.

*2.4.3. ROI analysis*

For all significant regions identified by the RFX model, independent activation peaks were confirmed in corresponding subject-specific ROIs, with very few exceptions at the threshold $p < 0.001$, uncorrected (t $>$ 3.45), and with no exceptions at the threshold $p < 0.01$, uncorrected (t $>$ 2.49). As for individual activation peaks found in ROIs that did not reach significance on the RFX model, on the other hand, the following was observed: There were only a very few spurious ROI peaks (1 additional peak found in 3 subjects, and 2 additional peaks found in 1 subject). However, there were three regions in which significant peaks, undetected by RFX analysis, were present in 29/32 subjects at the threshold $p < 0.001$, uncorrected (t $>$ 3.45), and in all subjects at the threshold $p < 0.01$, uncorrected (t $>$ 2.49). These were dorsal anterior cingulate gyrus (as a distinct peak from medial SMA), left posterior temporal lobe, and the left inferior parietal lobe (see Table 1b).

Another outcome of the ROI analysis concerns activation in the left inferior prefrontal cortex. In this region, independent activation peaks were identified by ROI analysis within a single activation cluster of the

Table 1a
Group-level random effects model activation foci ($p < 0.05$, FWE corrected) for the f-VFT

| Region (AAL) | MNI (x,y,z) | $t$-score |
|---|---|---|
| Medial supplementary motor area | −5, 14, 51 | 9.71 |
| Left inferior frontal gyrus/frontal operculum | −44, 32, 9 | 7.77 |
| Left precentral gyrus/*Premotor area | | |
| Superior | −30, −8, 65 | 7.95 |
| Inferior | −47, 5, 30 | 9.24 |
| Left thalamus | −10, 13, 13 | 7.01 |
| Left basal ganglia | −15, 5, 14 | 6.32 |

*Premotor area = Portions of superior and middle frontal gyri corresponding to BA 6.

Table 1b
Additional activation foci present in all 32 control participants as identified by ROI analysis of the f-VFT

| Region (AAL) | Average $t$-score (SD) |
|---|---|
| Dorsal anterior cingulate gyrus | 8.06 (1.87) |
| Left posterior temporal lobe | 5.78 (2.26) |
| Left inferior parietal lobe | 5.84 (1.89) |
| Left inferior frontal gyrus | |
| Pars opercularis | 8.32 (2.46) |
| Pars triangularis | 7.39 (1.73) |
| Left anterior insula | 5.81 (1.63) |

RFX model. Specifically, the ROI analysis revealed distinct peaks in the *pars opercularis* and *pars triangularis* portions of the inferior frontal gyrus and an additional peak in adjacent anterior insula.

## 3. Summary and discussion of control experiment findings

In this section, we described and tested methods for collecting fMRI data using a protocol that approximates traditional phonemically-based verbal fluency tests. The results from this experiment were highly consistent with results from previous fMRI studies that have investigated phonemic fluency [11]. Following methods introduced in Allen and Fong [43], we performed individual ROI analyses on each control subject. This analysis revealed two important details: First, activation peaks were confirmed on a subject-by-subject basis for each significant region identified by the group-level RFX analysis, with high consistency across subjects. Second, the ROI analyses revealed additional activation peaks in dorsal anterior ingulated, left posterior temporal lobe and left inferior parietal lobe, which were present in all subjects, but because the precise location of these peaks varied across subjects by a few millimeters, these peaks were not detected by the RFX analysis.

The primary objective of the ROI analysis, however, was to derive a distribution of sample $t$-values for each region activated in control subjects while performing the f-VFT. These regional distributions then allow us to perform quantitative assessments of individual patients. Altogether, our analyses identify 11 critical ROIs, including 6 regions identified by RFX and ROI analyses concurrently, as well as 3 additional regions, and a break-out of 3 additional peaks within the LIFG, identified by ROI analysis alone. These regions are presented in Table 2, along with the means and standard deviations computed from the maximal $t$-scores extracted from each of the 32 participants.

The data presented in Table 2 are useful for two reasons. First, the intensities of the means allow one to estimate relative activity levels within in each ROI during f-VFT performance, which might be interpreted as the relative contribution of each area toward performing the task. Conclusions of this sort, of course, must be drawn with some caution, given that some cortical regions are inherently prone to higher BOLD signal detection, all things being equal. Second, the variance of peak activations across individuals provides the basis for clinical assessment of individual patients. That is, the variance within each ROI is a numerical expression of the "normal" range of expected peak values, which provides a statistical means for evaluating activation levels from individual subject/patients. A practical ap-

Table 2
Means and standard deviations of extracted maximum t-values for each critical ROI associated with the f-VFT for a sample of 32 control subjects

| Region | Dorsal anterior cingulate | Medial SMA | Left inferior frontal gyrus *PO* | Left inferior frontal gyrus *PT* | Left inferior precentral gyrus | Left anterior insula | Left basal ganglia | Left inferior parietal lobe | Left posterior temporal lobe | Left thalamus |
|---|---|---|---|---|---|---|---|---|---|---|
| Group mean | 8.06 | 8.44 | 8.32 | 7.39 | 8.11 | 5.81 | 4.54 | 5.84 | 5.78 | 5.66 |
| Group StDev | 1.87 | 1.71 | 2.46 | 1.73 | 1.99 | 1.63 | 1.66 | 1.95 | 2.26 | 2.35 |

SMA = Supplementary Motor Area; *PO* = *Pars Opercularis*; *PT* = *Pars Triangularis*.

proach for applying this procedure is described in the next section.

## 4. Clinical application of the ROI analysis

In Allen and Fong [43] we used fMRI norms from our matrix reasoning protocol (the f-MRT) to assess a sample patient. The primary benefit of that analysis is that it demonstrated the power of fMRI to reveal details about the integrity of the independent neural mechanisms that underlie a cognitive task which extend beyond observations of task performance alone. Moreover, information about this region-by-region functional evaluation is provided in a format that is familiar to clinicians, that is, as a single value, score, label, or index (e.g., a $z$-score).

In this study, we describe a patient (CHB) with a clinical profile somewhat similar to the one reported in Allen and Fong [43]. The objective of this case study is to explore additional issues concerning the clinical application of our method. Specifically, we aim to demonstrate the discriminative power of the functional protocols reported in these two papers (the f-MRT and the f-VFT) within a single patient. CHB was chosen specifically because his cognitive deficits are highly selective in nature, with impairments limited almost exclusively to verbal processing skills. It is expected, therefore, that our fMRI protocols will be sensitive to this selectivity, given that the f-VFT and f-MRT are construed as predominately verbal versus nonverbal tests, respectively. In sum, to the extent that this patient shows selective impairments on traditional methods of assessment, we expect the same degree of selectivity in deviations from normal patterns in his fMRI data.

### *4.1. Patient description*

CHB is a 26 year-old married Caucasian male who was involved in a MVA approximately two years prior to study. The patient reported a positive loss of consciousness, with anterograde amnesia of 24 hours and approximately 24 hours of retrograde amnesia. Quantitative brain analysis based on T1 MRI imaging one- and two-years post injury revealed no abnormality. The patient reports primary difficulties with attention and concentration, memory impairment, word-finding problems, mood dysregulation, and loss of motivation.

A full neuropsychological battery two years post injury indicated global cognitive performance to be in the average to high average range for his age, gender, and level of education, except for measures of verbal memory and verbal fluency, which were in the low average to borderline impaired range. This marked contrast between verbal and nonverbal functioning is evident, for example, by CHB's performance on subscales of the Test of Memory and Learning [36]. His average index scores for verbal and nonverbal subtests were 95 and 127, respectively, with a significant difference at the $p < 0.01$ level. Details concerning his performance on the specific matrix reasoning and verbal fluency subtests he was administered are provided below.

### *4.2. Relevant subtests from conventional neuropsychological tests*

For patient CHB, scores were available for two variants of the matrix reasoning test and one variant of the phonemic verbal fluency test. Additionally, scores were available from a semantic fluency test. Although the semantic fluency test has been shown to activate a slightly distinct complex of anatomical regions from phonemic fluency, the two tests show strong functional overlap in most respects [11].

#### *4.2.1. Matrix reasoning*

CHB was administered the 26-item matrix reasoning test from the Wechsler Adult Intelligence Scale – Third Edition (WAIS – III). His performance was in the superior range, with a raw score of 24, equivalent to an age-based scaled score of 16, placing him at +2 SD above the mean (98 percentile) for this subtest. CHB was also administered the 36-item Raven's Colored Progressive Matrices test. He scored 100% on this test.

*4.2.2. Verbal fluency*

CHB was administered a verbal fluency test for the letters F, A, and S, with a time-limit of 60 sec per letter and standard exclusion instructions. His performance was in the low average range. He produced an average of 26.5 words (after correction for 2 errors), for an age- and education-adjusted score of $-1.63$ SD below the normative mean [41]. CHB was also administered the semantic fluency subtest of the Repeatable Battery for the Assessment of Neuropsychological Status – RBANS [34], which uses a single target category of *fruits and vegetables* with a 60 second time-limit. On this test he generated 17 words (with one error), placing his performance at -1.51 SD below the normative mean for his age range [35].

## 4.3. Patient CHB: fMRI assessment

In addition to conventional neuropsychological testing, CHB was concurrently administered a series of six fMRI protocols, including the f-MRT and the f-VFT. The activation results from these latter two tests are reported here.

### 4.3.1. Matrix reasoning: The f-MRT

*4.3.1.1. Methods*

The design and procedures for the f-MRT are identical to those described for control subjects in Allen and Fong [43]. Image acquisition and processing were likewise identical. First, functional images were submitted to a time-series ANCOVA model. The resulting $t$-score map was then overlain on CHB's coregistered and ROI-parcellated SPGR image. ROI cluster peak analysis was performed in the same manner as described for control subjects.

*4.3.1.2. Results*

CHB's responded correctly to 24/24 (100%) of the items on the f-MRT. His mean response time was 3056 ms (SD $=$ 1103 ms), which is about 350 ms faster than the normative group mean. In terms of functional activation, the ANCOVA model revealed a robust pattern of activation resembling the group RFX analysis of Allen and Fong [43], with significant peaks in all 9 cortical regions identified by that analysis.

*4.3.1.3. ROI analysis*

Data from CHB's ROI analysis confirm this apparent consistency with the control group, in terms of the size and distribution of his activation peaks. CHB's activation peaks occurred in all and only the significant ROIs of normative group. All $t$-values fell within 1 standard deviation of the mean, with an average *z-score* of $+0.15$ (see Fig. 2). It is important to note that the group mean values reported in Fig. 2, are not the mean values from the pooled RFX model, but rather are the means (and standard deviations) of subject-independent values extracted during ROI analysis.

### 4.3.2. Verbal fluency: The f-VFT

*4.3.2.1. Methods*

The design, procedures, image acquisition, and processing of the f-VFT for patient CHB were identical to those described for the control subjects in Experiment 1. Functional images were submitted to a time-series ANCOVA model, with the resulting $t$-score map overlain on CHB's coregistered and ROI-parcellated SPGR image for ROI cluster analysis.

*4.3.2.2. Results*

Consistent with the verbal nature of this task, CHB showed a left hemisphere dominant pattern of activation with foci in areas associated with lexical retrieval, lexical form selection, and covert articulation. However, CHB failed to show activation in several regions significant at the group level. The ROI analysis presented below details the nature of these discrepancies.

*4.3.2.3. ROI analysis*

As shown in Fig. 3, CHB showed weak or absent activation in most ROIs, except in left inferior parietal lobe, where his $t$-score was nearly one standard deviation above the norm. For all other ROIs, CHB's $t$-values fell more than 1 standard deviation below the mean (for 6 ROIs) or 2 standard deviations below the mean (for 2 ROIs), with an average $z$-score of $-1.47$ across all regions.

## 4.4. Patient CHB: Summary and general discussion

The assessments presented here on patient CHB show consistency between neuropsychological data and fMRI data. The agreement between these two methods increases our confidence that fMRI can be used as a practical and valid assessment tool at the single-patient level. At the very least, fMRI might be construed as a supplemental tool to conventional methods. However,

| Region | Fusiform/ Inferior Occipital Cortex | Middle Occipital Gyrus | Superior Parietal Lobule/ (IPS) | SMA/ Dorsal Anterior Cingulate | Thalamus | Precentral Gyrus | Anterior Insula | Superior/ Middle Frontal Gyrus | Basal Ganglia | Inferior Frontal Gyrus | Frontal Pole |
|---|---|---|---|---|---|---|---|---|---|---|---|
| |  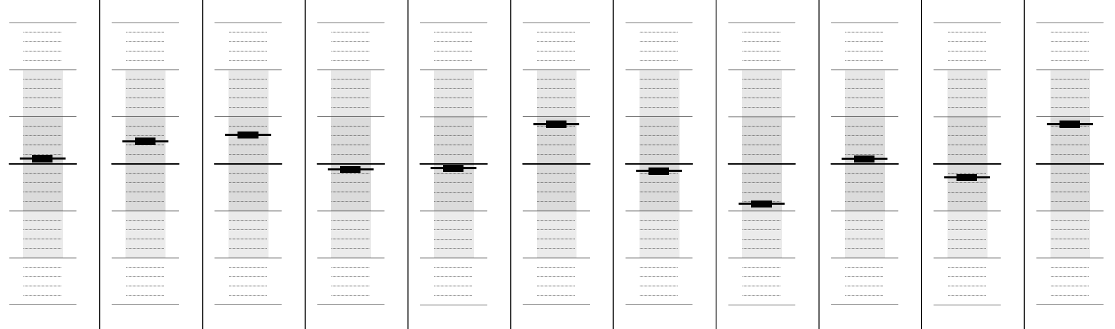 | | | | | | | | | | |
| Group Mean | 10.02 | 9.78 | 10.67 | 8.97 | 4.84 | 9.54 | 5.82 | 8.42 | 5.35 | 7.29 | 5.73 |
| Group StDev | 3.24 | 3.92 | 3.55 | 3.43 | 1.71 | 3.17 | 1.81 | 2.40 | 1.58 | 1.94 | 2.00 |
| Patient *t* value | 10.52 | 11.87 | 12.85 | 8.57 | 4.70 | 12.24 | 5.50 | 6.45 | 5.50 | 6.74 | 7.49 |
| Patient $z$-score | 0.16 | 0.53 | 0.61 | -0.12 | -0.08 | 0.85 | -0.18 | -0.82 | 0.10 | -0.28 | 0.88 |

IPS = Intraparietal Sulcus; SMA = Supplementary Motor Area

Fig. 2. ROI peak $z$-scores for patient CHB compared to the control group for the f-MRT. matrix reasoning test. Tick marks along vertical columns indicate patient $z$-scores ($-3$ to $+3$) for each brain region. Note: value levels do not simply represent relative activation in each region. Rather, they represent degree of statistical deviation from normal for each region.

fMRI offers unique advantages. For example, when a deficit is identified by conventional assessment, fMRI often provides further details about the nature of that deficit. Consider the data from CHB on the verbal fluency test above. His performance on the behavioral task, by itself, indicates only that he is impaired on that ability. However, the verbal fluency task, like most cognitive activities, requires the combined, integrated success of multiple processing systems. Minimally, the task requires lexical retrieval mechanisms within the language processing system as well as executive mechanisms responsible for such things as guiding memory search, holding previously selected words in working memory, and inhibiting word repetitions and other inappropriate responses. One might ask, then, which cognitive system(s) is/are responsible for CHB's impairment. His fMRI data suggest that both systems are compromised, as indicated by low activation in areas associated with lexical retrieval/production (left inferior frontal and posterior temporal areas) as well as areas associated with executive components of the task (dorsal AC and SMA).

CHB's profile in this respect contrasts with other patients with mild to moderate cognitive impairment following traumatic brain injury selected from among 100+ patients who have been tested on our protocol. For example, a male patient slightly younger than CHB had nearly the same performance as CHB on the FAS test ($z-$score $= -1.09$). However, this patient's fMRI pattern, following ROI analysis as described above, differed from CHB's. Like CHB, this patient also showed deficient activation in left inferior frontal and posterior temporal areas. However, unlike CHB, this patient's activation in medial frontal areas fell well within the normal range. Yet another patient, a female somewhat older than CHB (42 yrs), performed poorly on the VFT test ($z$-score $= -1.56$), but showed an opposite pattern. In contrast to CHB , this patient showed normal activation in left frontal/temporal areas, but like CHB, she showed subnormal activation in medial frontal cortex. Although space limitations preclude full discussion of further patients, the contrastive activation profiles evident in the brief descriptions of these 3 patients illustrate the potential for revealing distinct patterns of deficit within the set of neurological sub-systems that underlie a complex cognitive task, such as verbal fluency, using fMRI.

## 5. Summary and conclusion

Together with the study of Allen and Fong [43] we have addressed the emerging need to provide neuropsy-

| Region | Dorsal Anterior Cingulate | Medial SMA | Left Inferior Frontal Gyrus *PO* | Left Inferior Frontal Gyrus *PT* | Left Inferior Precentral Gyrus | Left Anterior Insula | Left Basal Ganglia | Left Inferior Parietal Lobe | Left Posterior Temporal Lobe | Left Thalamus |
|---|---|---|---|---|---|---|---|---|---|---|

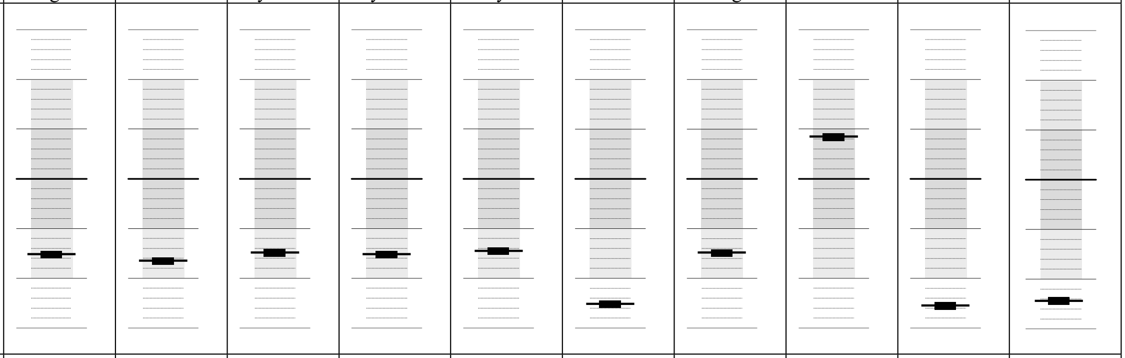

| Region | Dorsal Anterior Cingulate | Medial SMA | Left Inferior Frontal Gyrus *PO* | Left Inferior Frontal Gyrus *PT* | Left Inferior Precentral Gyrus | Left Anterior Insula | Left Basal Ganglia | Left Inferior Parietal Lobe | Left Posterior Temporal Lobe | Left Thalamus |
|---|---|---|---|---|---|---|---|---|---|---|
| Group Mean | 8.06 | 8.44 | 8.32 | 7.39 | 8.11 | 5.81 | 4.54 | 5.84 | 5.78 | 5.66 |
| Group StDev | 1.87 | 1.71 | 2.46 | 1.73 | 1.99 | 1.63 | 1.66 | 1.95 | 2.26 | 2.35 |
| Patient *t* value | 5.27 | 5.67 | 4.70 | 4.75 | 5.29 | 1.66 | 2.10 | 7.49 | 0.00 | 0.00 |
| Patient *z*-score | -1.49 | -1.62 | -1.47 | -1.53 | -1.42 | -2.54 | -1.47 | 0.85 | -2.55 | -2.41 |

SMA = Supplementary Motor Area ; *PO = Pars Opercularis*; *PT = Pars Triangularis*

Fig. 3. ROI peak z-scores for patient CHB compared to the control group for the f-VFT. This contrasts sharply with CHB's profile in Fig. 2.

chological assessments adapted for use with fMRI technology. We have presented here an adaptation of a phonemic verbal fluency test, the f-VFT, and shown that in a sample population of normal subjects, activation patterns are consistent both with previous fMRI studies using similar protocols, and activation is consistent with cognitive mechanisms hypothesized to be critical for successful performance on the phonemic VFT in terms of underlying cortical structures.

As such, we suggest that the data presented in these studies greatly advance the usefulness of fMRI as a diagnostic and assessment tool, particularly in that we provide protocols that yield outcomes structured in such a way that performance and activation patterns to be compared in meaningful ways to performance on conventional assessments that are familiar to most clinicians (e.g., as illustrated by the visual reports in Figs 2 and 3). We envisage the greatest value of these efforts, however, as providing a means for collecting and archiving activation patterns from normal subjects, such that the reliability and locations of activation peaks can be evaluated and catalogued on a region-by-region basis across subjects. This procedure yields a range of expected "normal" activation values for each region, and thus provides a normative scale for evaluating individual patient outcomes.
